# Runoff events and related rainfall variability in the Southern Carpathians during the last 2000 years

**DOI:** 10.1038/s41598-019-41855-1

**Published:** 2019-03-29

**Authors:** Jack Longman, Daniel Veres, Vasile Ersek, Aritina Haliuc, Volker Wennrich

**Affiliations:** 1 Department of Geography and Environmental Sciences, Northumbria University, Newcastle-upon-Tyne, NE1 8ST United Kingdom; 2 School of Ocean and Earth Sciences, University of Southampton, National Oceanography Centre, Waterfront Campus, Southampton, SO14 3ZH United Kingdom; 3 Romanian Academy, Institute of Speleology, Clinicilor 5, 400006 Cluj-Napoca, Romania; 4 Research Institute of the University of Bucharest, University of Bucharest, 050107, Bucharest, Romania; 5 Institute of Geology and Mineralogy, University of Cologne, 50674 Cologne, Germany; 6 Present Address: School of Geography and the Environment, University of Oxford, South Parks Rd, Oxford, OX1 3QY United Kingdom; 7 Present Address: Charles University, Faculty of Mathematics and Physics, Department of Atmospheric Physics, Ke Karlovu 3, Prague, 121 16 Czech Republic

## Abstract

The occurrence of heavy rainfall events is expected to undergo significant changes under increasing anthropogenic forcing. South-eastern Europe is reacting rapidly to such changes, therefore understanding and forecasting of precipitation variability is vital to better comprehending environmental changes in this area. Here we present a sub-decadal reconstruction of enhanced rainfall events for the past 2000 years from the Southern Carpathians, Romania using peat geochemistry. Five clear periods of enhanced rainfall are identified at 125–250, 600–900, 1050–1300, 1400–1575 and 1725–1980 CE. Significant runoff is observed during the second half of the Medieval Warm Period, whilst the Little Ice Age was characterised by significant variability. The North Atlantic Oscillation appears to be the main control on regional precipitation, but changes in solar irradiance also seem to play a significant role, together with the Siberian High. Comparison of the data presented here with model outputs confirms the ability of models to predict general trends, and major shifts, but highlights the complexity of the region’s hydrological history.

## Introduction

Understanding the causes and effects of wetter periods and associated hydro-meteorological events (heavy precipitation, flooding) is of great interest in climate science^1^, as the majority of modelling studies predict higher incidence of such events in the near future^2^. Heavy precipitation usually leads to flooding, the risk to human life, and an increase in economic disturbances^1,3,4^. However, comprehension of the spatial and temporal distribution, as well as the magnitude and driving mechanisms of high rainfall, runoff and flooding events is still incomplete. South-eastern Europe is one of the most rapidly reacting areas to current climate change^2,5^ and to understand the long-term hydroclimate variability in this region, high-resolution and well-dated records are needed. Within eastern Europe, projections suggest a shift of North Atlantic storm tracks northward, which will lead to more extreme weather patterns^6,7^, with higher risk of droughts^2^, and stronger cyclonic activity^8^. Since cyclonic activity is one of the major controls on northern hemisphere precipitation variability^6,9^, understanding its long-term behaviour and environmental impact is vital. However, projections are extremely uncertain, with models unable in most cases to reliably resolve storm track movements without validation against long-term observational data^10^.

For the most recent past, and especially the last century, data on rainfall variability in the Carpathian area, and its linkage to atmospheric pressure systems may be inferred from documentary sources^11–13^, and meteorological observations^14,15^. So far, in order to asses long-term hydrological changes and disentangle past rainfall variability research focused mainly on geochemical records of peat bogs and lake sediments from central-western Europe^16–22^. With some exceptions, comparable high-resolution, well-dated palaeohydrological records from eastern Europe are rare^23–26^, particularly records at yearly, or near-yearly resolution^27^.

Compared to the hydrology of central-western Europe, which is primarily influenced by Atlantic forcings, meteorological observations^14,28,29^ and proxy data^25,26,30^ show south-eastern Europe is periodically subjected to the North Atlantic, Siberian and Mediterranean atmospheric pressure systems. As a result, local to regional climate is seasonally and sometimes abruptly affected by shifts in these systems^23,30–32^. In particular, shifts in the strength of North Atlantic Oscillation (NAO) have been linked to changes in winter precipitation^14,28,33^, and winter temperature in the region^14^. Several paleoenvironmental records appear to confirm this connectivity has persisted for the past few millennia^23,25,34,35^. However, other records, particularly from the eastern Mediterranean, show no clear link to the NAO^36,37^. As such, further high-resolution palaeoclimate data from south-eastern Europe may strengthen our understanding of long-term patterns and amplitudes of change in the pressure systems mentioned above, as well as their environmental impact across the continent as a whole^30^.

**Figure 1:**
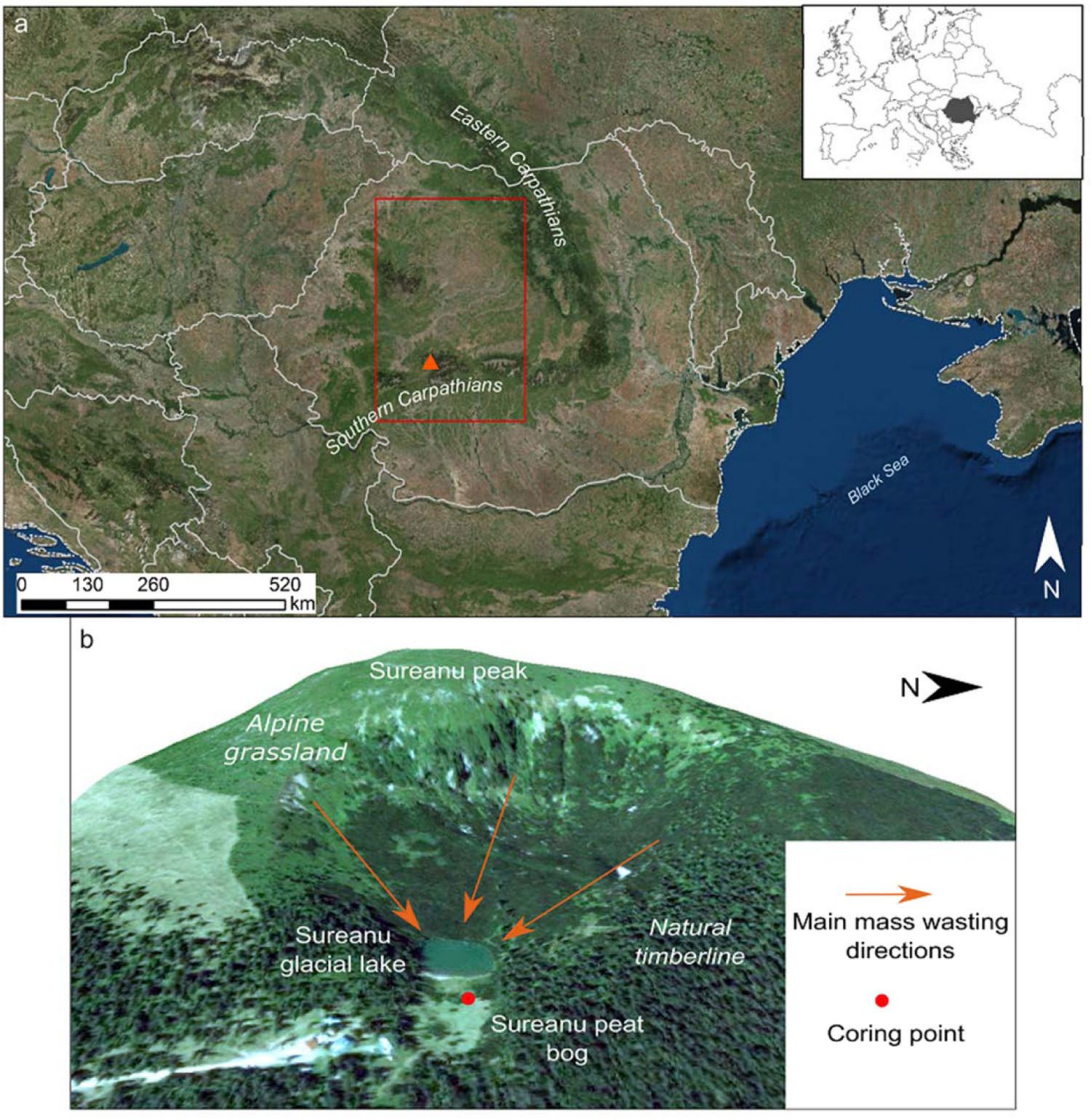
Location of Sureanu peat bog. Upper panel displays the location Sureanu (red triangle) within Romania, with the red rectangle indicative of the region from which model data was derived (Map created in ArcMap 10.3, Environmental Systems Resource Institute, ArcMap 10.3 ESRI, Redlands, California, http://desktop.arcgis.com/en/arcmap/). Lower panel shows 2D satellite image of the area with the location of the bog, lake and direction of mass wasting events. For scale, lake is 75 m across. Geomorphological features such as avalanche channels and eroded areas are clearly visible. (Imagery source: Esri, DigitalGlobe, GeoEye, Earthstar Geographics, CNES/Airbus DS, USDA, USGS, AeroGRID, IGN, and the GIS User Community).

Here we present a new high-resolution geochemical record of sediment input onto Sureanu bog (Southern Carpathians, Romania, Fig. 1) linked to precipitation variability in the catchment area, and associated runoff erosion for the last 2000 years. Such work builds on a previous, low-resolution study into the paleoclimatic history of this area, which demonstrated a clear link between variations in lithogenic element concentrations and land erosion.

## Results and Discussion

**Figure 2:**
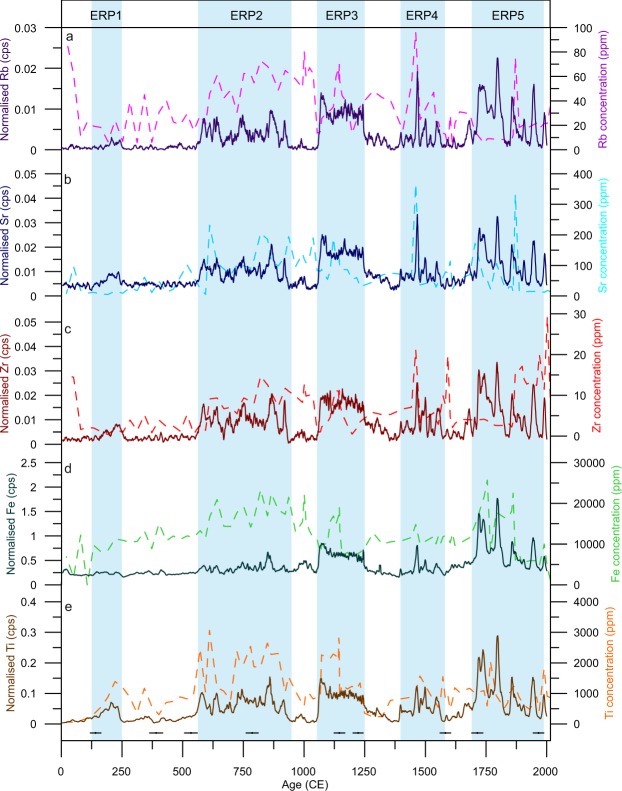
Lithogenic element data from Sureanu peat bog. Displayed here are ITRAX-derived normalised lithogenic element counts (solid lines), and ICP-derived concentrations (dashed lines). Blue rectangles denote periods of enhanced runoff (ERPs) as mentioned in text. Also displayed are radiocarbon dates, and errors, as used to construct the age model (Black circles).

**Figure 3:**
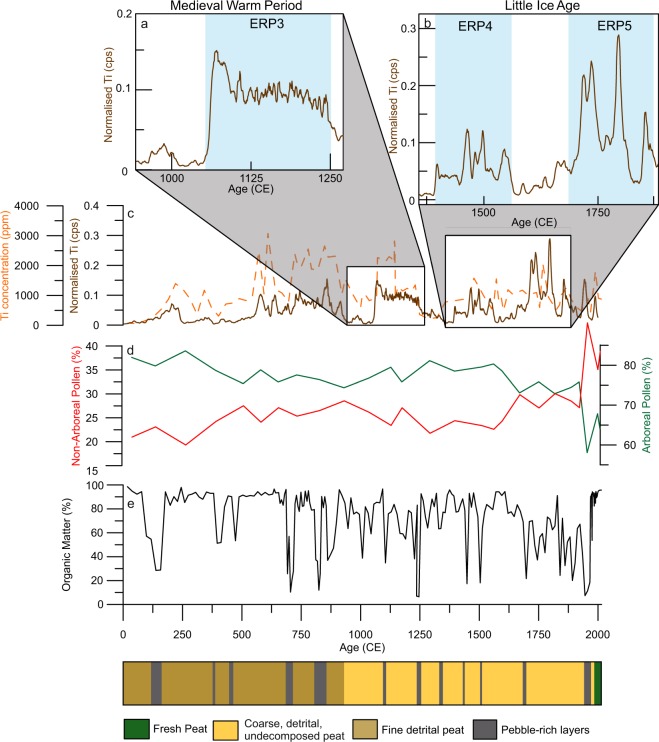
ITRAX normalised Ti counts (panel c), and ICP-derive Ti concentration as in Fig. 2, used as a proxy for changing precipitation, with closer focus on the Medieval Warm Period (**a**) and Little Ice Age (**b**). Blue rectangles as in Fig. 2. Additionally (**d**), percentages of all arboreal taxa and all non-arboreal taxa from Sureanu are presented^25^. Also displayed (**e**) is the lower resolution record of organic matter content within Sureanu bog^25^, and a simplified lithological diagram.

When interpreting X-Ray Fluorescence Core-Scanning (XRF-CS) data, particularly with peat sediment, it is important to ensure the variations truly reflect changes in depositional geochemistry^38^. To ensure the XRF-CS data discussed here provide a reliable method of geochemical screening for the Sureanu peat record, comparison of ITRAX results with those from destructive Inductively Coupled Plasma Optical Emission Spectrometry (ICP-OES) has been made (Fig. 2). Visually, the XRF-CS data reflects the broad changes observed in the ICP-derived geochemical series (Fig. 2). By comparing the ICP and the XRF-CS data on the same age scale (at 20 year resolution), statistical comparisons may be attempted. Positive correlations significant at the 0.01 level (p-value < 0.001) may be observed for Fe (r^2^ = 0.209), Sr (r^2^ = 0.301) and Ti (r^2^ = 0.496) whilst for Zr the correlation is significant at the 0.05 level (r^2^ = 0.106, p-value = 0.037). These correlations, although low, are considerable when the constraints of the two methods are considered. ICP analysis provides the elemental concentration of a 1cm-wide sediment slice, and therefore encompasses 5 ITRAX data-point measurements. Further, ITRAX screening analyses only the uppermost fraction of the sample surface, and results may be impacted by variations in water content and surface roughness. Finally, the fact that analysis via ICP requires bringing the sample into solution introduces another uncertainty, that of digestion. Whilst all attempts have been made, including the addition of HF to ensure total digestion, it may not be possible in all cases. For Rb no such correlation is observed, likely a result of Rb concentrations approaching the ITRAX detection limit. However, correlation between all XRF-CS-derived datasets (Table 1) suggests records of all lithogenic elements may be used to reconstruct runoff related erosion in Sureanu peat bog’s catchment area (Fig. 2). For ease of comprehension, however, we focus here on the Ti record (Fig. 3e).

**Table 1:** Cross-correlation values for all normalised ITRAX counts of lithogenic elements.

	Fe	Rb	Sr	Ti	Zr
Fe	n/a	0.8164	0.7774	0.8975	0.7720
Rb		n/a	0.7791	0.9376	0.7812
Sr			n/a	0.8241	0.8383
Ti				n/a	0.8383
Zr					n/a

Sureanu peat bog is located at the base of a glacial cirque and is surrounded on three sides by steep slopes (Figs 1b and S1^25^). These slopes display numerous mass-wasting related geomorphological features, including erosional channels, debris lobes, and exposed rock (Figs 1b and S1, S2^39^). This means that mass-wasting activity and superficial runoff affecting the slopes will likely be channelled towards Iezerul Sureanu Lake and the adjacent Sureanu bog. Furthermore, any overtopping of the lake during highstands will normally result in mineral debris being washed onto the bog. Longman *et al*. 2017 discussed a number of mechanisms (e.g. avalanches, debris flows, runoff, or lake flooding) that may result in mass-wasting activity and the deposition of minerogenic matter (MM) onto Sureanu bog^25^, but the common feature is their linkage with high precipitation events. The resolution of the record means that each year is covered by one or two data points, and so should be considered indicative of all erosion occurring at the site in the previous half-year or one year. As a result, the rate at which MM is exported from the slope to the bog is inconsequential, and the record should not be considered indicative of individual events, but of an average value indicative of all erosion occurring in the preceding period of time. By whatever specific mechanism, periods of increased MM deposition within Sureanu bog may be considered representative of increased precipitation in the catchment area, and thus stronger runoff.

The comparability of all lithogenic elements as analysed via ITRAX indicate similar controls on their deposition (Fig. 2), and a dominant and geochemically distinct mineral source. This contrasts with what would be expected if trends in the lithogenic elements reflect various local and/or distal lithogenic sources, including significant input of long-distance transported dust. In such cases, shifts in relative geochemical makeup of the depositional events should be observed in different lithogenic elements, linked to, for example, changing (proximal to distal) dust sources^40–42^. Furthermore, typical average grain sizes within the MM-rich layers at Sureanu are larger (40–130 μm^25^) than would be expected from distal atmospheric dust deposition during the Holocene (~20 μm)^43^. Contribution of distal dust, as observed within Mohos peat bog in the Eastern Carpathians^40^ cannot be ruled out, but this is clearly overprinted by the contribution from local rocks at Sureanu. Evidence for such events inputting MM into the bog are visually documented, particularly in the uppermost 100 cm of the record, with a number of detrital layers, some containing pebbles >2 mm, observable^25^ (Fig. 3).

Erosional regimes are not dictated solely by precipitation, with human activity and especially deforestation potentially enhancing erosion by removing protective vegetation. However, there is evidence at Sureanu^25^, and other records in the Southern Carpathians^44^ that the onset of deforestation had begun near Sureanu earlier than the onset of regular minerogenic deposition. Additionally, the fact that steep slopes encircle the bog makes the area unsuitable for grazing (Figs 1b and S1), and its location at the upper limit of the treeline (Supplementary Fig. S2), means that purposeful deforestation for timber or pasturing has not been strong. Indeed, pollen data (Fig. 3 and Supplementary Figure 3), indicates there is no association between the observed increases in lithogenic element concentration, and decreased forest cover as inferred from arboreal taxa percentages. Further, no anthropic pollen taxa appear to increase in concentration during periods of high runoff (Supplementary Fig. S3), suggesting there is no relationship between greater human impact on the landscape, and greater erosion at this particular site. As such, we confidently infer that the periods with enriched lithogenic element concentrations are indicative of increased regional rainfall and runoff, with limited or no anthropic contribution except perhaps the last decades. There are five such major periods identifiable in the record (Fig. 2): 125–250 CE, 600–900 CE, 1050–1300 CE, 1400–1575 CE and 1725–1980 CE, hereafter referred to as Enhanced Runoff Periods (ERP).

The first ERP identified in our record for the last 2000 years has been dated between c. 125–250 CE (Fig. 2) which is in agreement with previous reconstructions indicating wetter conditions at this time^45–47^ in the Carpathian area, although other records suggest drier conditions^23,48^. After 600 CE, the Sureanu bog record indicates an extended period of minerogenic input onto the bog, persisting until roughly 900 CE, and representing the longest period of continual lithogenic input in the record (ERP 2). Therefore, we assume this is representative of increased rainfall and runoff at Sureanu throughout this time. The resolution of the Sureanu record allows for the identification of specific peaks in rainfall/runoff within this period, with 625 ± 64 CE and 825 ± 93 CE appearing to display particularly wet conditions. Higher frequency erosional events may be linked with the Dark Ages Cold Period (600–900 CE), characterised in south-eastern Europe by increased rainfall as documented in a wide range of high-altitude palaeohydrological reconstructions^48,49^. Furthermore, Carpathian fluvial archives document regular flooding during this time^50,51^ suggestive of increased regional runoff and moisture availability.

In the Sureanu record, two distinct phases are discernible within the MWP. The first, 900–1060 CE, is characterised by low values for all lithogenic elements (Figs 2, 3) and likely reflects a period of low precipitation. After 1050 CE, a rapid and clear increase in the lithogenic elements occurs up to 1300 CE, likely reflecting a rise in regional precipitation^23,24,49^ and denoted here as ERP 3 (Fig. 3a). High precipitation for this period is paralleled by an increase in *Fagus*^25^, which thrives in warm and wet conditions^52^, and a decrease in *Alnus* observed at this time in the Sureanu pollen record^25^. Such distinct two-phase hydrological variability during MWP has not been documented in other regional climate reconstructions, perhaps due to coarser temporal resolution of these records. Studies indicate dry conditions in western Romania^53,54^, but other proxy data provide contrasting reconstructions, with testate amoeba-derived records indicating wet conditions throughout much of the MWP^49,55^. This is in line with observations of increased erosional regimes^23,35^, reflecting a trend seen Europe wide^18,56–58^. Other than the shift observed at 1060 CE, the Sureanu record documents few variations (Fig. 3a), suggesting the MWP was likely moderately wet, but with relatively low variability as observed elsewhere^31,54^. Certainly when compared to the variability (both in terms of sharp fluctuations, and in amount of runoff and minerogenic input onto the bog) of later periods in the record (Fig. 3a), the MWP is not particularly distinct in the Sureanu archive.

The moisture availability that characterised the late MWP as reconstructed from the Sureanu record gradually decreased from 1250 CE onwards, reaching its nadir by 1300 CE, with dry conditions persisting until 1400 CE (Fig. 2). Limited runoff activity is reflected in the minerogenic matter record^25^, where limited lithogenic input has been documented at this time. This also explains the decrease in *Fagus* pollen at 1300–1400 CE at Sureanu^25^, as dry conditions are sub-optimal for this taxa^52^. This is reflective of region-wide dry conditions^23^, and relatively low hydroclimate variability, in contrast to western European records, where a number of studies document high rainfall in the period between MWP and the Little Ice Age (LIA)^18,20,57,58^.

After 1400 CE, a return to wetter conditions (ERP 4) may be inferred (Fig. 3), with the timing suggesting this shift is coeval with the onset of Little Ice Age (LIA). The LIA, a period of continent-wide cool temperatures, is characterised in the Sureanu record by distinct fluctuations in minerogenic input (Fig. 3b). Of these, two periods show the most significant variability at 1400–1575 CE and 1725–1980 CE, respectively. The resolution of our record allows for an unprecedented investigation into the extent of wet periods during and post LIA in this region. Our data indicate that such fluctuations in minerogenic input onto the bog could be linked to episodic decadal-length peaks in rainfall, separated by similarly long periods of low minerogenic input, and thus low precipitation. Variability in moisture availability during the LIA in eastern Europe has been observed previously^49,59^, but the available analytical resolution did not allow for observing the fine details of LIA hydroclimate variability as seen in the Sureanu record (Fig. 3b).

From 1400–1575 CE, three main peaks in minerogenic input are discernible, centred on 1465 ± 103, 1490 ± 91 and 1550 ± 85 CE (Fig. 3b). These data suggest the early LIA was characterised by higher and variable moisture availability, echoing results from other studies^24,60^, but that wet conditions were not characteristic of the whole early LIA. This is followed by evidence of a prolonged dry period in the Sureanu record, between 1550–1715 CE (Fig. 3b). This agrees well with other proxy reconstructions of hydroclimate variability, with very dry spells identified in eastern Carpathian peat records between 1550–1750 CE^49,61^, and after 1580 CE^60^.

**Figure 4:**
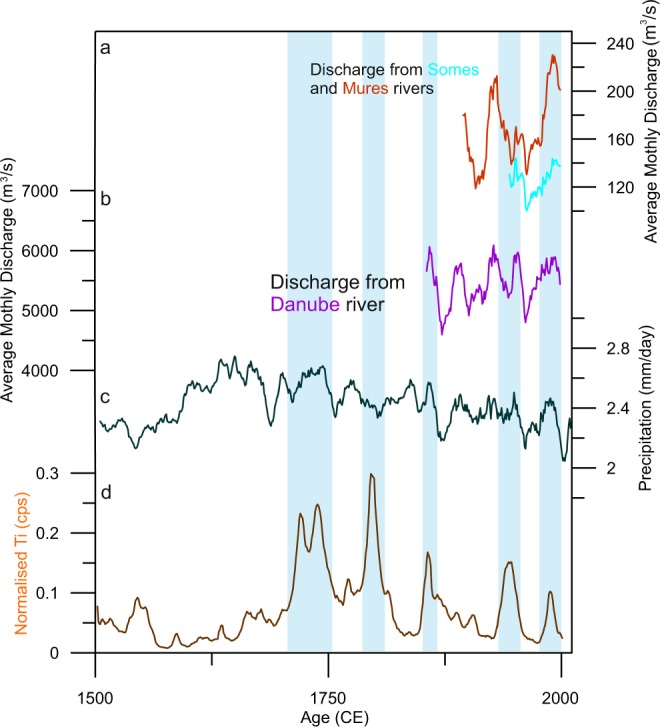
Comparison of Ti-derived precipitation at Sureanu (**d**) with river discharge values from Romanian rivers, the Somes and Mures (**a**) and Danube (**b**). Also presented is gridded precipitation data for the area 23°–24°E, 45°–46°N^89^.

This period of low moisture availability is followed by a sharp transition into the most variable section of the record (ERP 5), between 1725–1980 CE, encompassing the largest peaks in lithogenic elements (Fig. 3)^25^. As a result, these minerogenic-rich peaks may be considered representative of the wettest interval in the record, echoing the cold and wet summers recorded across Europe^62^, with the variability representative of high-frequency oscillations observed at other sites in central and eastern Europe^61,63,64^. This instability appears symptomatic of the spatial hydroclimate variability in the broader central-eastern Europe region (including the Carpathians) during the LIA. In northern Romania, wetter conditions were not observed until after 1780 CE, whilst in south-west Romania, regular droughts are seen in the 18^th^ Century^65^. Persistent dry conditions are reconstructed for northern Romania after ~1550 CE based on testate amoeba^24,60^. The high precipitation period between 1780–1815 CE, appears linked to several regionally representative high river discharge events, with documentary evidence attesting to a number of major floods in the period 1784–1785 CE^66^, with limited model results corroborating wet conditions^36^ (Fig. 4).

**Figure 5:**
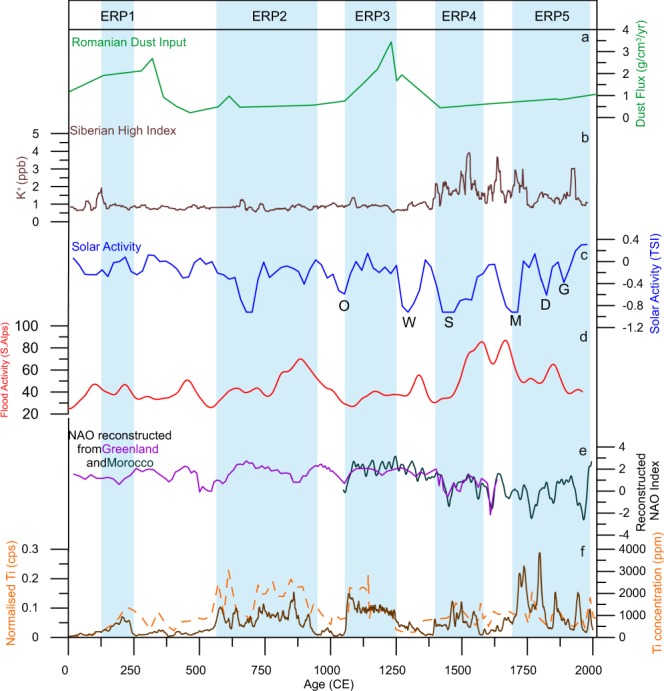
Comparison of (panel f) normalised Ti counts, and Ti concentration from Sureanu with forcings, and other palaeohydrological records. Displayed here is (**a**) dust input to a Romanian peat bog in the eastern Carpathians^40^, (**b**) Siberian High Index as derived from K^+^ from Greenland ice^102^, (**c**) solar activity^73^ and the timing of the Oort (O), Wolf (W), Spörer (S), Maunder (M), Dalton (D),Gleissberg (G) minima. Also displayed is (**d**) the record of flood activity from the southern Alps^18^, and (**f**) North Atlantic Oscillation (NAO) as reconstructed from Greenland^103^ and Morocco^104^.

Disagreements between central and eastern European palaeohydrological records have been observed before^23^, likely reflecting the north-south^67^ and east-west^37^ gradients in European hydroclimate. Particularly in the LIA, the Sureanu record displays notable correspondence with flooding and precipitation records in the Alpine region^18^, where evidence suggests high levels of rainfall, occurring in notable pulses^17,22^, as at Sureanu (Fig. 5). As such, it appears the Late Holocene Sureanu record largely reflects a similar variability in Atlantic derived rainfall, but modulated also by input from the Mediterranean as recently reconstructed for south-eastern Europe in a comparison between speleothem stable isotope data and regional pollen records^68^.

After 1900 CE there are two other periods of enhanced runoff in the Sureanu record (Fig. 2). The first spans the middle of the 20^th^ Century (centred on 1935 ± 98 CE), whilst the second occurs during the century’s end, peaking by 1978 ± 65 CE (Fig. 2). The end of the first period may reflect a decrease in rainfall observed across Romania roughly 1969 CE^28^, reflected also in river discharge data (Fig. 4). However, the lack of similarity outside of this time to other regional drought indices^65^ and rainfall records may be indicative of erosion linked to increased local human activity, as ski slopes, and touristic activity became significant in the area^25^. What is clear from the most recent section of the Sureanu record is the increase in the prevalence of enhanced runoff in the last 500 years. Whether this variability reflects solely a higher incidence of extreme precipitation events or increasingly stronger human impact in the area requires further research.

To understand the forcing mechanisms behind the hydroclimatic variability reconstructed here, we compare the Sureanu record to a number of regionally representative climatic indices potentially linked to the variability observed (Fig. 5). It has been speculated by a number of studies that the North Atlantic Oscillation (NAO) should be the most important atmospheric pressure system influencing hydroclimatic variability in eastern Europe^15,25,69^. This is because during periods of NAO positive phase (NAO^+^), intensification of the westerlies shifts Atlantic storm tracks northwards. This means Atlantic derived moisture is distributed mainly in the north and west of the continent, whereas moisture over south-eastern Europe is derived primarily from the Mediterranean sea^15,31^. Conversely, when the NAO is in negative phase (NAO^−^), weak westerlies allow for the Atlantic storm tracks to move eastwards, resulting in increased Atlantic-derived winter precipitation over south-eastern Europe^25,31^. From the comparison in Fig. 5, it appears that the NAO variability controls some aspects of the Sureanu runoff record, with four periods of enhanced precipitation directly ascribable to NAO^−^ conditions, and some short-term fluctuations, especially those within the LIA, also potentially linked to a negative NAO phase.

**Figure 6:**
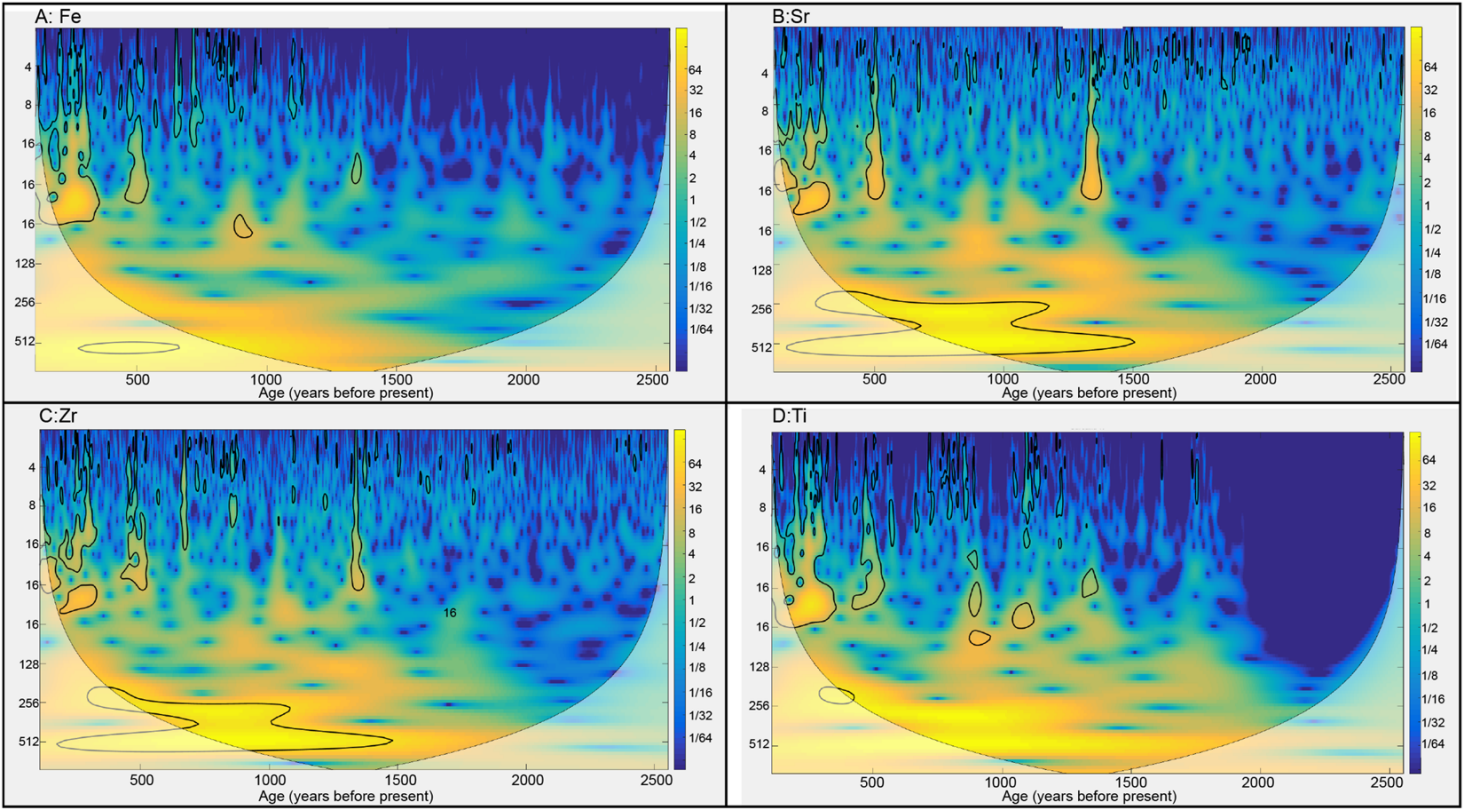
Spectral analysis of Sureanu ITRAX geochemical data for a: Fe, b: Sr, c: Zr and d: Ti. Areas outlined in black are significant at the 95% confidence level. Shaded areas indicate the cone of influence, outside which results may be unreliable.

**Figure 7:**
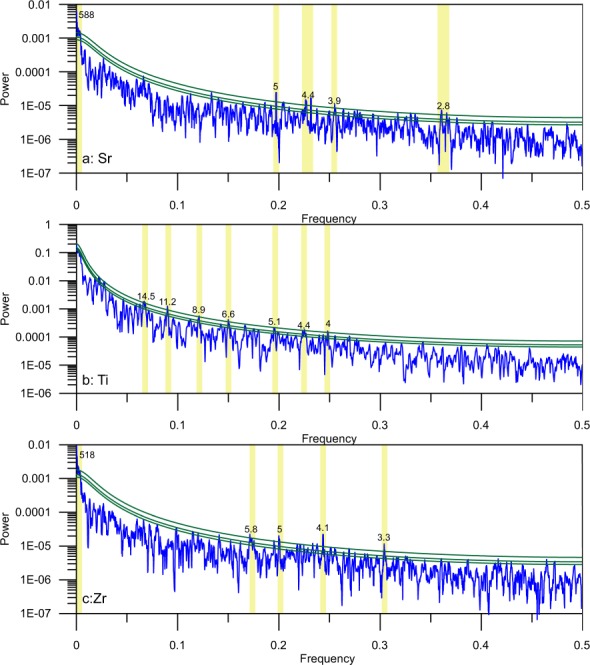
Multi-Taper Method (MTM) power spectrum of Sureanu ITRAX geochemical data for a: Sr, b: Ti and c: Zr. Green lines signify the 90%, 95% and 99% confidence levels. Above the 99% level, signals have been interpreted, and are highlighted with yellow rectangles.

The primary low-frequency cycles observed in the Sureanu record (200 and 500-year, Fig. 6) do not appear to correlate with Atlantic climatic fluctuations, and so may be linked to another forcing mechanism. Further, the lack of any correlation with the Atlantic Multidecadal Oscillation, which has a cyclicity of 60–80 years^70^, suggests it plays a limited role in the hydroclimate system of eastern Europe as reconstructed from the Sureanu record. However, short-term oscillations as observed via the multi-taper method (MTM) could be linked to the NAO, with results displaying persistent 5-year and 4-year cycles in all elements analysed (Fig. 7). Similar cycle lengths have been observed previously in NAO-controlled varve sedimentation^70^, so it is possible that NAO variability is playing a distinct role in driving the small-scale, short-duration minerogenic deposition cycles identified in the Sureanu data.

Meteorological evidence suggests however that winter precipitation regimes over Romania are actually controlled by the combination of the NAO, Scandinavian Pattern (SCA), and the East Asia-West Russia (EA-WR) pattern^33^, whilst the impact of the changing strength of the Siberian High (SH) has also been suggested to further modulate the regional hydroclimate variability^71^. Interestingly, the onset of LIA in the Sureanu record (around 1400 CE), characterised by increased precipitation at this time (Fig. 3), occurs during a strengthening of the SH (Fig. 5). Disentangling the impact of the SH on most recent hydroclimate variability over south-eastern Europe, however, requires further research. In the case of the LIA and more recent minerogenic record at Sureanu, after apparent pacing of its onset by a strong SH, further peaks appear linked to NAO^−^ conditions, whereas others (e.g., ~1740 and ~1950 CE) agree well with periods of stronger SH conditions (Fig. 5). This echoes previous work on flooding in the eastern Mediterranean, which displays a similar link between flooding and SH strength in the Late Holocene^72^. This evidence points to the fact that interplay between the two mechanisms is, at least periodically, and particularly in the past 500 years, modulating the signal observed at Sureanu. This observation clearly displays that a denser network of records is necessary for better disentangling the interplay between these circulation modes and their long-term environmental impact in the wider Eurasian region.

In addition to atmospheric pressure systems, periods of low solar irradiance, known as grand minima, have been linked to colder and wetter climates^73–75^. Noticeable similarities between the reconstructed runoff variability at Sureanu and solar activity may be observed (Fig. 5). The onset of the wet phase in the MWP occurred during the Oort minimum, whilst the first wet interval within the LIA occurred during the Spörer minimum, and later runoff events occur during the Dalton and Gleissburg minima (Fig. 4). This reflects well existing data from various studies in which wet conditions have been linked to solar minima, with such evidence across Europe^63,76,77^, and North America^78^. Further evidence of the impact of solar irradiance cycles on the Sureanu runoff record may be observed in the existence of 500-year and 200-year cycles in much of the lithogenic geochemical data (Fig. 5), and in the MTM power spectrum for Zr and Sr (Fig. 7). Such cycles may be linked to solar irradiance, with a 500-year cycle (a harmonic of the postulated Hallstatt cycle) observed in radiocarbon records^79^, and in flooding archives from central Europe^18^. The 200-year cycle observed in the last 1000 years of the Sureanu record (Fig. 6) is most likely representative of the Seuss cycle^79,80^. This oscillation has been noted previously in peat archives^40,81^, and in marine records from the Baltic Sea^82^.

Changes in solar radiation may influence trophospheric circulation through downward propagation of planetary waves^83^, particularly impacting atmospheric circulation pattern over the North Atlantic, and the influence it exerts on Eurasian hydroclimate variability. Therefore, the postulated influence of solar irradiance upon the atmospheric pressure systems (explaining why the two appear to fluctuate in tune) and their interaction appear to be the main driver behind past runoff variability observed in Sureanu record.

To further investigate the inferred past runoff record from Sureanu based on proxy-data, and the ability of climate models to address past regional climate fluctuations, we compare the Ti-derived record with modelled precipitation values for the past 2000 years, using the model output from PaleoView^84^, which utilises climate data from the TRaCE21ka experiment^85,86^. This experiment uses the simulated projections from Community Climate System Model version 3 (CCSM3^87,88^), a coupled atmosphere-ocean general circulation model. This model resembles present-day regional and global temperature and hydrology data and may therefore be suitable for addressing climate variability over spatially large areas, and for long-term comparisons.

**Figure 8:**
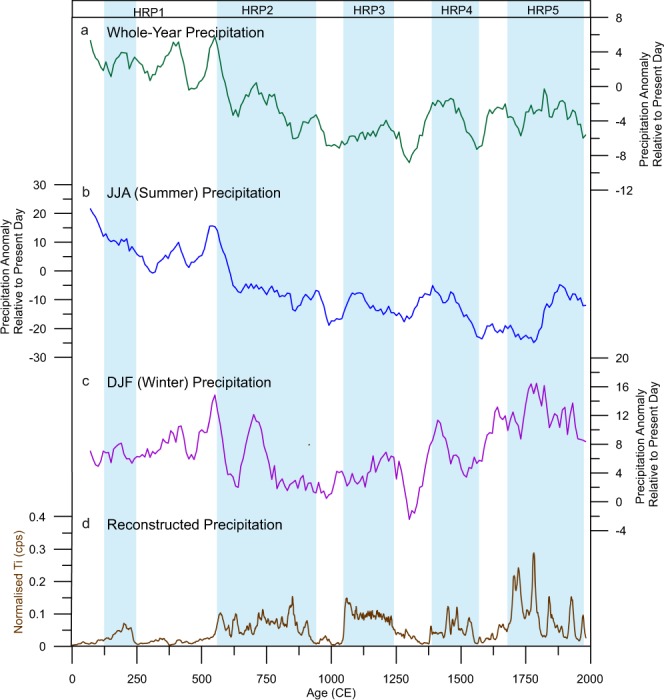
Comparison of model output from the study region with observation-derived Sureanu rainfall data. Presented here are three model scenarios; (panel a) whole-year, (**b**) summer (JJA) and (**c**) winter (DJF). These are displayed alongside smoothed, normalised Ti-derived erosion from Sureanu (**d**). Blue rectangles are as displayed in Fig. 2.

Figure 8 shows a comparison between data from three model scenarios (year-long, winter (DJF) and summer (JJA) averages) of the grid cell Sureanu is located in 45–47.5°N, 22.5–25°E, for central-eastern Europe (35–50 °N, 15–30 °E) and the Ti-derived record from Sureanu peatbog. Model data is presented in anomaly form, relative to the present day^84^. To allow for comparison, all data was brought onto the same 10-year interval timescale (using a 30-year Gaussian window) (Fig. 8). For all three scenarios, the Sureanu runoff data suggests a discrepancy in the amount of rainfall for the first 500 years (0–600 CE) of the compared sections when compared to model reconstructions (Fig. 8). Model data suggests this period is the wettest, whilst our reconstructed runoff data indicate low runoff, particularly between 250–600 CE. This discrepancy is clearest in the JJA and whole-year model output, whilst the DJF output for this time period is much closer to the signal observed in the XRF-CS data (Fig. 8). This lack of comparability for both JJA and whole-year model data continues throughout much of the record, although some increases in erosion observed toward the end of the record are reproduced by the model, with model-related precipitation increases observable between 1400–1600 CE and around 1800 CE. However, for most of the period these two model outputs bear little resemblance to the measured data. For the DJF model output, however, more similarities may be observed (Fig. 8). The three most recent periods of enhanced runoff at Sureanu parallel the periods of increased runoff, between 1100–1250 CE, 1350–1600 CE and 1700–1850 CE, with concurrent drops in modelled precipitation also present between these peaks. This apparent agreement indicates the Sureanu runoff record may be related primarily to winter precipitation, at least during this part of the record. Since winter precipitation over central-eastern Europe is generally linked to NAO fluctuations, this may explain why the Sureanu record, when comparing to proxy data reflecting atmospheric pressure systems, bears most relation to the NAO (Fig. 5). Considering the uncertainties of the model, for the last 500 years, we compare our dataset with annual precipitation reconstructed for the area 23°–24°E, 45°–46°N^89^, while for the very recent interval, spanning the past 150 years, we look at river discharge data (National Center for Atmospheric Research, 2001) (Fig. 4)^90^. For the wetter period centred on 1780 CE, it appears that Sureanu record agrees with reconstructed precipitation^89^ (Fig. 4c). In addition, the latest period of higher rainfall, at the end of the 20^th^ Century appears to resemble peaks in discharge data.

Despite the apparent ability of the data to reflect regional hydrological trends, it is clear the model does not echo such data for much of the record, with periods of non-alignment, particularly in the earliest section of the record. Precipitation and other hydroclimatic indexes are complex, localized (dependent on topography and fine-scale processes) parameters^36,91^. Further, the coarse resolution of the model means the small-scale fluctuations recorded via river-discharge records, and the Sureanu record, are not reproduced. Therefore, such parameters are difficult to predict via models, and as a result the periods of good correspondence may be promising indicators of the improving nature of climate models. As such, further high-resolution records of palaeoprecipitation in the region are needed to constrain the modelling results. Approaches such as the one performed here allow for the presentation of high-resolution data and will allow for greater accuracy when developing model parameters in the future.

## Conclusions

We present the first record of sub-decadal fluctuations in runoff activity in the Southern Carpathians, Romania for the last 2000 years using XRF-CS and ICP-OES derived lithogenic elemental data. Our data provide a high resolution record of palaeohydrological variability for this region, documenting five periods of enhanced runoff (125–250 CE, 600–900 CE, 1050–1300 CE, 1400–1575 CE and 1725–1980 CE). These periods generally agree well with published reconstructions but provide an unprecedented high-resolution insight into short-term fluctuations. In particular, the Medieval Warm Period was characterised by an early dry phase with limited runoff, followed by a wet, but stable phase after 1050 CE. The Little Ice Age is characterised by the most variable precipitation regime of the entire record, with the largest peaks in runoff proxies, indicative of the wettest conditions.

Our data suggests the NAO is the dominant control on Southern Carpathian hydroclimate, but that, as previously suggested, other controls, such as the Siberian High may play a role in moderating the NAO’s regional impact. Further, there appears to be a strong link between solar irradiance minima and the onset of wet periods, itself likely linked to the NAO, particularly during the past 1000 years. Data-model comparison appears to confirm the apparently strong impact of the NAO. The comparability of model-derived precipitation values for winter (DJF) to the erosion-related record suggest it primarily records input of lithogenic material following winter precipitation. The lack of similarity to summer and annual precipitation appears to confirm this. Despite an apparent link between model and observed data, particularly in the last 1000 years, there appears to be a disconnection for the period 0–1000 CE, where very few similarities may be observed. Such a conclusion indicates the necessity of further high-resolution studies into palaeohydrology in this region, in order to better constrain model predictions.

## Methods

### Site description

Sureanu peat bog (45°34.05′N, 23°30.28′E), is a small bog located at the foot of a glacial cirque (gradient in excess of 1 in 2) in the Southern Carpathians^25^. The bog is uncovered, hydrologically linked to the neighbouring glacial tarn Iezerul Sureanu (Figs 1 and S1, S2), and at the uppermost forest limit in this area^92^, at 1840 m above sea level. As such, the vegetation on the surrounding slopes is sparse, consisting of subalpine taxa, typically grasses, shrubs (especially *Juniperus*) and dwarf pine (*Pinus mugo*). Numerous mass-wasting related geomorphological features may be observed (Fig. 1c), including avalanche channels. Climatically, the area is temperate continental, with typical average winter temperatures between −2 °C and −7 °C, and between 8–19 °C in summer^93^. Typically, precipitation amounts to between 900–1800 mm per year, mainly as snowfall during winter months. The site, therefore, is under snow cover for between 100–200 days per year^93^. Because of the bog’s location at the foot of a glacial cirque, any debris dislodged from surrounding slopes during periods of slope erosion is deposited onto its surface, thus disturbing peat growth. The reconstruction of such deposition therefore may be used as a proxy for changes in erosion and linked to rainfall variability^25^.

Two separate cores were extracted from the bog centre using a Russian peat corer^25^. The first core (SUR-1, 603 cm long) was wrapped in clingfilm, and transported to Northumbria University, where it was stored at 3 °C prior to subsampling for Inductively Coupled Plasma-Optical Emission Spectrometry (ICP-OES) analysis. The second core (SUR-2) was transported to the University of Cologne for X-Ray Fluorescence-Core Scanning (XRF-CS) analysis. As both cores were located next to one another, the correlation between them was established via alignment of geochemical data.

### Geochemical methods

XRF-CS was performed on the uppermost 200 cm of SUR-2 record, using an ITRAX core scanner equipped with an Si drift detector^94^ at the University of Cologne (Institute of Geology and Mineralogy), Germany. Measurements were made at 2 mm intervals, with 20 s count times at each interval. Analysis was performed using a Cr X-Ray tube set to 30 kV and 50 mA. From the wide range of elements analysed, only lithogenic elements (Fe, Rb, Sr, Ti, Zr), which display high enough concentrations above the detection limits were selected for further interpretation. For ease of interpretation, a 9-point running average has been applied to the raw XRF-CS data. To ensure various issues associated with XRF-CS analysis of peat, including high but variable organic content, variable density, and changing water content are taken into account, the raw cps values have been normalised with respect to total (incoherent + coherent) scattering^40,95^.

To further test whether the ITRAX data reliably reproduced the geochemical signal, ICP-OES analysis of the same lithogenic elements was carried out on samples collected at roughly 2 cm intervals. These samples were brought into solution using a mixed acid (HNO_3_-HCl-HF) digestion^25^, prior to analysis via a Perkin Elmer Optima 8000 system at Northumbria University. An internal standard (either 1ppm In or 1ppm Sc) was measured to correct for instrumental drift, whilst two certified reference materials (Montana Soil 2711 and IAEA Lake Sediment) were run alongside samples, with recoveries documented in Supplementary Table S1. Blanks, with negligible contamination for all elements were also run at regular intervals. We directly compare our data with the loss on ignition-derived record of minerogenic deposition^25^.

### Chronology

The age model is based upon 9 radiocarbon dates, analysed on bulk peat samples from the uppermost 200 cm of core SUR-1^25^. Analysis was carried out via accelerator mass spectrometry at HEKAL AMS Laboratory, MTA ATOMKI Institute for Nuclear Research of the Hungarian Academy of Sciences in Debrecen, and at the ^14^C Centre at Queen’s University Belfast. Using the IntCal13 Calibration curve^96^, and Bacon software, an age-depth model was developed, indicating 200 cm of sediment documents just over 2000 years of deposition (Supplementary Fig. S4).

### Time series analysis

To identify non-stationary cyclicities in the proxy data, continuous Morlet wavelet transform was used^97,98^. The Morlet wavelet is commonly employed in analysing geophysical data and produces a two-dimensional frequency-time mapping of the data, allowing identification of quasiperiodic oscillations in time series with multiple frequencies. To allow for this analysis, the normalised lithogenic element data were interpolated to equal 1-year time steps using a Gaussian window of 3 years. To further analyse the data, the Multi-Tapered Method (MTM) was performed^99,100^. The MTM does not have an *a priori* assumption about the processes producing the time series that are analysed, and is a useful technique for analysing time series which could have both continuous and singular components^101^. For ease of interpretation, only datasets displaying significant cyclicities are displayed here.

## Supplementary information

Supplementary Information

Dataset 1

## Data Availability

All ITRAX-derived data is uploaded as supplementary information. Other datasets generated during and/or analysed during the current study would be provided on request.
