# Supplementary material for: Runoff events and related rainfall variability in the Southern Carpathians during the last 2000 years
Source: Sci Rep. 2019 Mar 29;9:5334. doi: 10.1038/s41598-019-41855-1 (PMC6440959; doi:10.1038/s41598-019-41855-1)
Supplement: Supplementary file 1 — Supplementary Information [file 41598_2019_41855_MOESM1_ESM.docx]

**Supplementary Information for “Runoff events and related rainfall variability in the Southern Carpathians during the last 2000 years”**

Jack Longman^1^, ^2^, ^6*^, Daniel Veres^3^, Vasile Ersek^1^, Aritina Haliuc^4,7^, Volker Wennrich^5^

^1^ Department of Geography and Environmental Sciences, Northumbria University, Newcastle-upon-Tyne, NE1 8ST, United Kingdom

^2^ School of Ocean and Earth Sciences, University of Southampton, National Oceanography Centre, Waterfront Campus, Southampton, SO14 3ZH, United Kingdom

^3^ Romanian Academy, Institute of Speleology, Clinicilor 5, 400006 Cluj-Napoca, Romania

^4^ Research Institute of the University of Bucharest, University of Bucharest, 050107 Bucharest, Romania

^5^ Institute of Geology and Mineralogy, University of Cologne, 50674 Cologne, Germany

^6^ Present address: School of Geography and the Environment, University of Oxford, South Parks Rd, Oxford OX1 3QY, United Kingdom.

^7^ Present address: Charles University, Faculty of Mathematics and Physics, Department of Atmospheric Physics, Ke Karlovu 3, 121 16, Prague, Czech Republic

^*^ Corresponding author. Jack Longman. Email: jack.longman@ouce.ox.ac.uk

**Supplementary Figures**


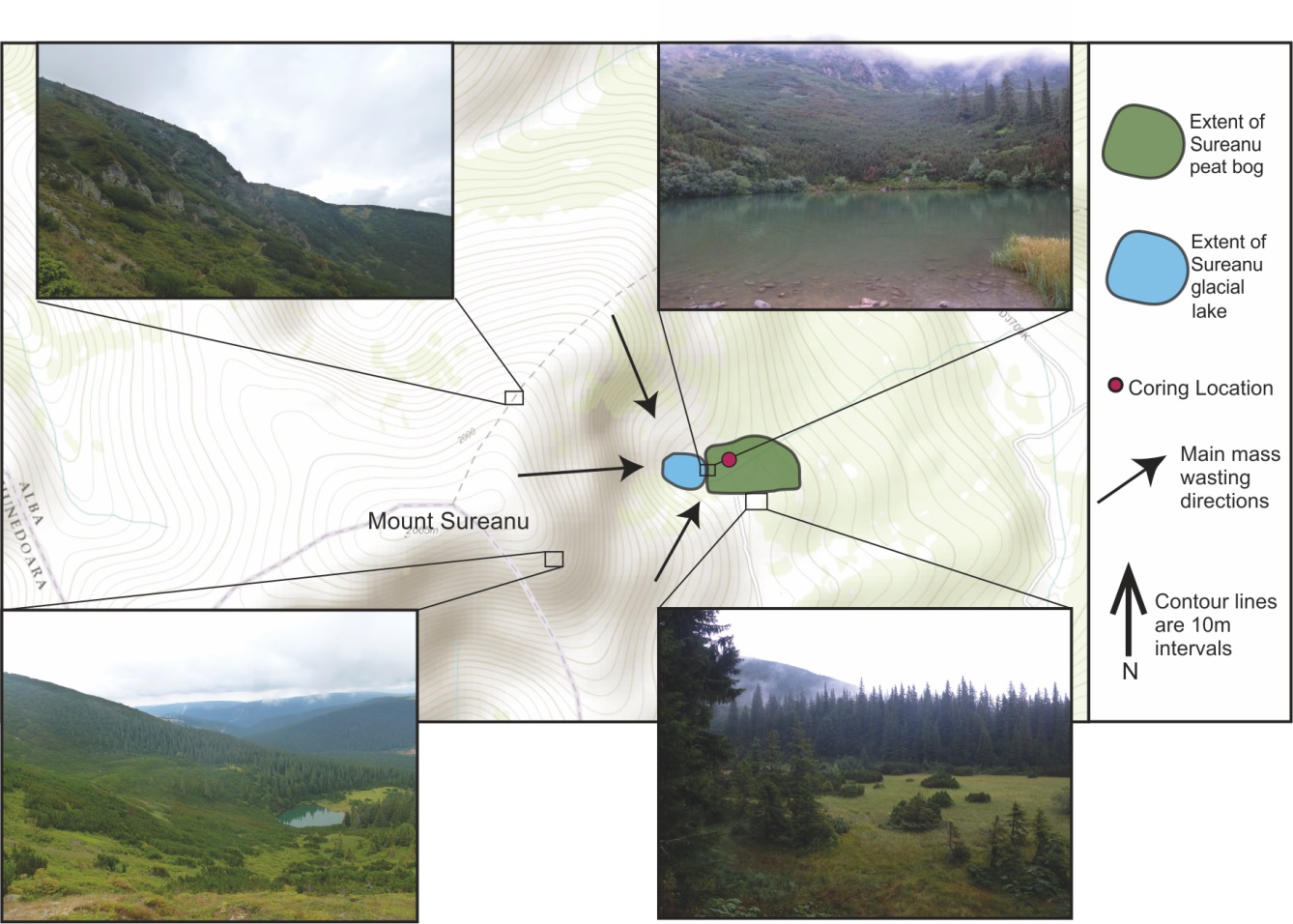


Supplementary Figure S1: Topography of the study site, with the location of the bog and lake indicated. Additionally, photographs of the site, and surrounding slopes are included, along with their location.


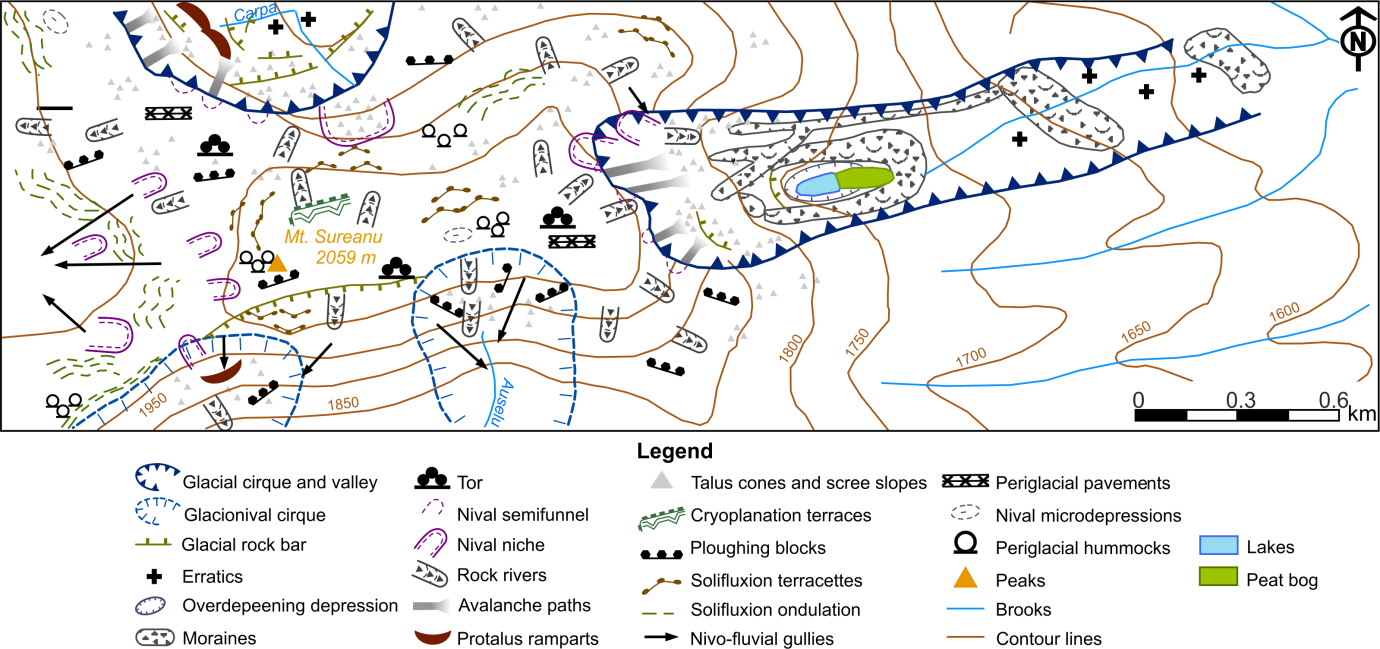


Supplementary Figure S2: Geomorphological map of Sureanu area (redrawn after Urdea and Reuther, 2009 ^1^)


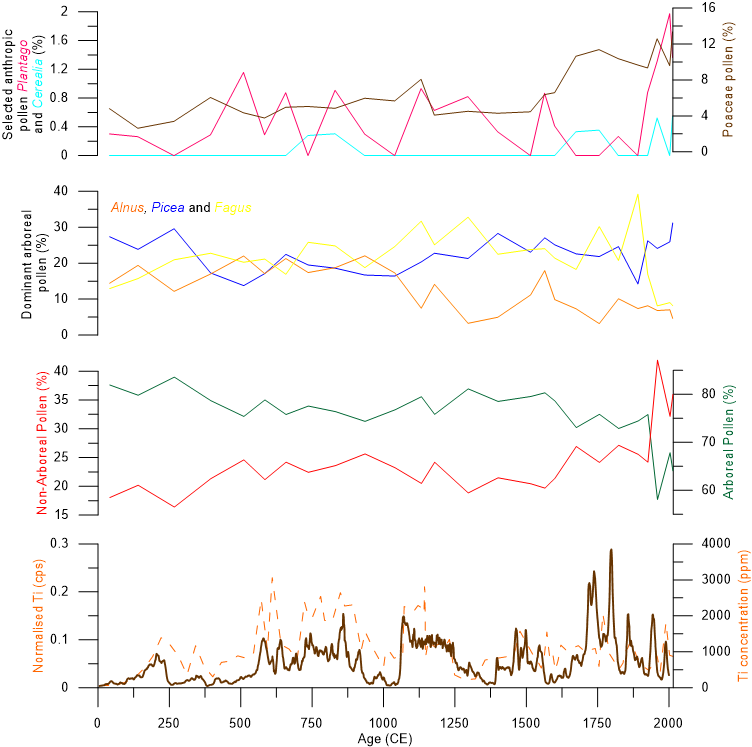


Supplementary Figure S3: Comparison of selected pollen data with the Ti-based reconstruction of enhanced runoff periods. Data from ^2^.


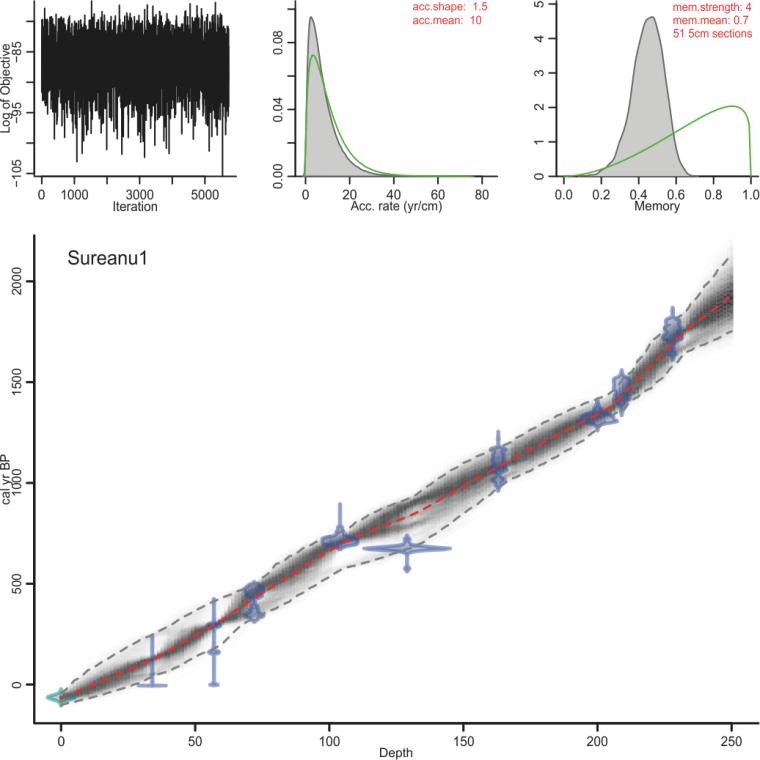


Supplementary Figure S4: Age model from the uppermost section of core SUR-1.

**Supplementary Tables**

Table 1: Supplementary Table S1: Measured certified reference material (CRM) values for each element analysed.

| **Montana Soil 2711a** | **Fe** | **Rb** | **Sr** | **Ti** | **Zr** |
| --- | --- | --- | --- | --- | --- |
| Rpt 1 | 26880 | 142.5 | 202.1 | 3158 | 207.1 |
| Rpt 2 | 26070 | 137.3 | 239.3 | 3188 | 201.2 |
| Rpt 3 | 24860 | 141.3 | 211.9 | 3300 | 209.5 |
| Rpt 4 | 25740 | 107.5 | 222.6 | 3055 | 200.2 |
| Rpt 5 | 27360 | 108.9 | 230.9 | 3015 | 200.6 |
|  |  |  |  |  |  |
| **Average** | 26182 | 127.5 | 221.36 | 3143.2 | 203.72 |
| Expected | 28900 | 110 | 245.3 | 3060 | 230 |
| **Recovery (%)** | 90.60 | 115.91 | 90.24 | 102.72 | 88.57 |
| Std Deviation | 978.58 | 17.73 | 14.79 | 112.95 | 4.28 |
| **RSD (%)** | 3.73 | 13.91 | 6.680 | 3.593 | 2.101 |
|  |  |  |  |  |  |
| **IAEA-SL-1 Lake Sediment** | **Fe** | **Rb** | **Sr** | **Ti** |  |
| Rpt 1 | 58950 | 135.2 | 101.5 | 5014 |  |
| Rpt 2 | 59630 | 110.3 | 91.3 | 4879 |  |
| Rpt 3 | 61500 | 128.5 | 91.48 | 4987 |  |
| Rpt 4 | 59590 | 120.5 | 97.14 | 4685 |  |
| Rpt 5 | 65250 | 128.4 | 95.9 | 5205 |  |
|  |  |  |  |  |  |
| **Average** | 60984 | 124.58 | 95.464 | 4954 |  |
| Expected | 67400 | 113 | 80 | 5170 |  |
| **Recovery (%)** | 90.48 | 110.25 | 119.33 | 95.82 |  |
| Std Deviation | 2568.01 | 9.53 | 4.26 | 190.84 |  |
| **RSD (%)** | 4.21 | 7.64 | 4.464 | 3.852 |  |

**Supplementary References**

1. Urdea, P. & Reuther, A. U. Some new data concerning the Quaternary glaciation in the Romanian Carpathians. *Geogr. Pannonica* **13,** 41–52 (2009).

2. Longman, J., Ersek, V., Veres, D. & Salzmann, U. Detrital events and hydroclimate variability in the Romanian Carpathians during the Mid-to-Late Holocene. *Quat. Sci. Rev.* **167,** 78–95 (2017).
